# *Staphylococcus capitis* isolated from bloodstream infections: a nationwide 3-month survey in 38 neonatal intensive care units

**DOI:** 10.1007/s10096-020-03925-5

**Published:** 2020-06-09

**Authors:** Marie Decalonne, Sandra Dos Santos, Rémi Gimenes, Florent Goube, Géraldine Abadie, Saïd Aberrane, Vanina Ambrogi, Raoul Baron, Patrick Barthelemy, Isabelle Bauvin, Olivier Belmonte, Emilie Benabid, Rafik Ben Ammar, Salma Ben Hadj Yahia, Yasmina Berrouane, Philippe Berthelot, Alain Beuchee, Emmanuelle Bille, Pascal Bolot, Stéphanie Bordes-Couecou, Antoine Bouissou, Sandra Bourdon, Nadège Bourgeois-Nicolaos, Sophie Boyer, Christian Cattoen, Vincent Cattoir, Chantal Chaplain, Céline Chatelet, Aurore Claudinon, Nathalie Chautemps, Hélène Cormier, Céline Coroller-Bec, Benjamin Cotte, Carole De Chillaz, Olivier Dauwalder, Aude Davy, Martine Delorme, Maryvonne Demasure, Luc Desfrere, Michel Drancourt, Clarisse Dupin, Véronique Faraut-Derouin, Arnaud Florentin, Virginie Forget, Nicolas Fortineau, Tania Foucan, Pierre Frange, Karine Gambarotto, Géraldine Gascoin, Laure Gibert, Jacques Gilquin, Audrey Glanard, Jacqueline Grando, Alain Gravet, Jérôme Guinard, Geneviève Hery-Arnaud, Claire Huart, Nadia Idri, Jean-Marc Jellimann, Olivier Join-Lambert, Sylvie Joron, Philippe Jouvencel, Marie Kempf, Sophie Ketterer-Martinon, Mouna Khecharem, Serge Klosowski, Franck Labbe, Adeline Lacazette, Fabrice Lapeyre, Jérôme Larche, Peggy Larroude, Anne Le Pourhiennec, Nolwenn Le Sache, Sylvie Ledru, Annick Lefebvre, Clément Legeay, Florence Lemann, Claire Lesteven, Marion Levast-Raffin, David Leyssene, Isabelle Ligi, Alain Lozniewski, Pierre Lureau, Franck-Olivier Mallaval, Edith Malpote, Stéphane Marret, Pascale Martres, Guillaume Menard, Laura Menvielle, Laurent Mereghetti, Véronique Merle, Pascale Minery, Virginie Morange, Julien Mourdie, Anaelle Muggeo, Jean Nakhleh, Marie-Noëlle Noulard, Claude Olive, Hugues Patural, Pascale Penn, Manuel Petitfrere, Bruno Pozetto, Brigitte Riviere, Audrey Robine, Christine Roques Ceschin, Raymond Ruimy, Amine Siali, Stéphanie Soive, Souad Slimani, Anne-Sophie Trentesaux, Dominique Trivier, Christian Vandenbussche, Laurent Villeneuve, Evelyne Werner, Stéphane Le Vu, Nathalie Van Der Mee-Marquet

**Affiliations:** 1 SPIADI, CPIAS CVDL, Hôpital Bretonneau, Centre Hospitalier Universitaire, 37044 Tours, France; 2 Cellule d’Epidémiologie Régionale des Infections Nosocomiales, CPIAS CVDL, Service de Bactériologie-Virologie-Hygiène, Hôpital Trousseau, CHRU, 37044 Tours, France; 3 Service de réanimation néonatale, Centre Hospitalier Universitaire Félix Guyon, 97400 Saint Denis de la Réunion, France; 4 Laboratoire de Microbiologie, Centre Hospitalier Inter-Communal, 94010 Créteil, France; 5 Équipe opérationnelle d’hygiène, Centre Hospitalier Universitaire, 31059 Toulouse, France; 6 Équipe opérationnelle d’hygiène, Centre Hospitalier Universitaire, 29609 Brest, France; 7 Équipe opérationnelle d’hygiène, Hôpital de la Conception, APHM, 13005 Marseille, France; 8 Service de réanimation néonatale, Centre Hospitalier, 64000 Pau, France; 9 Laboratoire de Microbiologie, Centre Hospitalier Universitaire Félix Guyon, 97400 Saint Denis de la Réunion, France; 10 Équipe opérationnelle d’hygiène, Centre Hospitalier, 95300 Pontoise, France; 11 Service de réanimation néonatale, Centre Hospitalier Universitaire Antoine-Béclère, APHP, 92140 Clamart, France; 12 Laboratoire de Microbiologie, Centre Hospitalier, 62100 Calais, France; 13 Équipe opérationnelle d’hygiène, Centre Hospitalier Universitaire, 06200 Nice, France; 14 Équipe opérationnelle d’hygiène, Centre Hospitalier Universitaire, 42055 Saint Etienne, France; 15 Service de réanimation néonatale, Centre Hospitalier Universitaire, 35000 Rennes, France; 16 Laboratoire de Microbiologie clinique, Hôpital universitaire Necker-Enfants malades, APHP, 75015 Paris, France; 17 Service de réanimation néonatale, Centre Hospitalier Delafontaine, 93205 Saint Denis, France; 18 Équipe opérationnelle d’hygiène, Centre Hospitalier, 64100 Bayonne, France; 19 Service de réanimation néonatale, Centre Hospitalier Universitaire, 37044 Tours, France; 20 Équipe opérationnelle d’hygiène, Centre Hospitalier du Havre, 76290 Montivilliers, France; 21 Laboratoire de Microbiologie, Centre Hospitalier Universitaire Antoine-Béclère, APHP, 92140 Clamart, France; 22 Laboratoire de Microbiologie, Centre Hospitalier Universitaire Charles Nicolle, 76000 Rouen, France; 23 Laboratoire de Microbiologie, Centre Hospitalier, 59300 Valenciennes, France; 24 Laboratoire de Microbiologie, Centre Hospitalier Universitaire, 35000 Rennes, France; 25 Laboratoire de Microbiologie, Centre Hospitalier Delafontaine, 93205 Saint Denis, France; 26 Équipe opérationnelle d’hygiène, Centre Hospitalier, 62300 Lens, France; 27 Laboratoire de Microbiologie, Centre Hospitalier, 95107 Argenteuil, France; 28 Service de réanimation néonatale, Centre Hospitalier Métropole Savoie-Site de Chambéry, 73 011 Chambéry, France; 29 UPLIN, Centre Hospitalier Universitaire, 49933 Angers, France; 30 Équipe opérationnelle d’hygiène, Centre Hospitalier, 72000 Le Mans, France; 31 Clinique du Val d’Ouest, 69130 Ecully, France; 32 Service de Néonatalogie et Réanimation néonatale, Hôpital universitaire Necker-Enfants malades, APHP, 75015 Paris, France; 33 Laboratoire de Microbiologie, Hôpitaux Civils de Lyon, 69677 Bron, France; 34 Équipe opérationnelle d’hygiène, Centre Hospitalier, 22000 Saint Brieuc, France; 35 Équipe opérationnelle d’hygiène, Centre Hospitalier, 79021 Niort, France; 36 Équipe opérationnelle d’hygiène, Centre Hospitalier Régional, 45100 Orléans, France; 37 Service de réanimation néonatale, Centre Hospitalier Universitaire, Hôpital Louis-Mourier, APHP, 92700 Colombes, France; 38 Laboratoire de Microbiologie, Hôpital de la Conception, APHM, 13005 Marseille, France; 39 Laboratoire de Microbiologie, Centre Hospitalier, 22000 Saint Brieuc, France; 40 Équipe opérationnelle d’hygiène, Centre Hospitalier Universitaire Antoine-Béclère, APHP, 92140 Clamart, France; 41 Service d’hygiène et d’analyses environnementales (SHAE), Hôpitaux de Brabois, 54035 Nancy, France; 42 Équipe opérationnelle d’hygiène, Centre Hospitalier Métropole Savoie-Site de Chambéry, 73 011 Chambéry, France; 43 Équipe opérationnelle d’hygiène, Centre Hospitalier Universitaire, Kremlin Bicêtre, APHP, 94275 Le Kremlin Bicêtre, France; 44 Équipe opérationnelle d’hygiène, Centre Hospitalier Universitaire, 97159 Pointe-à-Pitre, France; 45 Équipe opérationnelle d’hygiène, Hôpital universitaire Necker-Enfants malades, APHP, 75015 Paris, France; 46 Équipe opérationnelle d’hygiène, Centre Hospitalier Universitaire Félix Guyon, 97400 Saint Denis de la Réunion, France; 47 Service de réanimation néonatale, Centre Hospitalier Universitaire, 49933 Angers, France; 48 Équipe opérationnelle d’hygiène, Centre Hospitalier, 81100 Castres, France; 49 Équipe opérationnelle d’hygiène, Centre Hospitalier Delafontaine, 93205 Saint Denis, France; 50 Équipe opérationnelle d’hygiène, Hôpitaux Civils de Lyon, 69677 Bron, France; 51 Laboratoire de Microbiologie, Centre Hospitalier, 68100 Mulhouse, France; 52 Laboratoire de Microbiologie, Centre Hospitalier Régional, 45100 Orléans, France; 53 Laboratoire de Microbiologie, Centre Hospitalier Universitaire, 29609 Brest, France; 54 Équipe opérationnelle d’hygiène, Centre Hospitalier, 59300 Valenciennes, France; 55 Équipe opérationnelle d’hygiène, Hôpital Louis-Mourier, APHP, 92700 Colombes, France; 56 Laboratoire de Microbiologie, Hôpital Louis-Mourier, APHP, 92700 Colombes, France; 57 Service de réanimation néonatale, Centre Hospitalier Universitaire, Hôpitaux de Brabois, 54035 Nancy, France; 58 Laboratoire de Microbiologie, Centre Hospitalier Universitaire, 14000 Caen, France; 59 Service d’hygiène, Centre Hospitalier, 62100 Calais, France; 60 Service de réanimation néonatale, Centre Hospitalier, 64100 Bayonne, France; 61 Laboratoire de Bactériologie-Hygiène Institut de Biologie en Santé, CRCINA Inserm U1232, Université d’Angers, Centre Hospitalier Universitaire, 49933 Angers, France; 62 Service de réanimation néonatale et réanimation pédiatrique, Centre Hospitalier Universitaire de Martinique, 97261 Fort de France, France; 63 Laboratoire de Bactériologie-Hygiène, Centre Hospitalier Universitaire, Kremlin Bicêtre, APHP, 94275 Le Kremlin Bicêtre, France; 64 Service de réanimation néonatale, Centre Hospitalier, 62300 Lens, France; 65 Laboratoire de Microbiologie, Centre Hospitalier du Havre, 76290 Montivilliers, France; 66 Service de réanimation néonatale, Centre Hospitalier Universitaire, 97159 Pointe-à-Pitre, France; 67 Service de réanimation néonatale, Centre Hospitalier, 59300 Valenciennes, France; 68 Polyclinique Saint Roch, 34000 Montpellier, France; 69 Équipe opérationnelle d’hygiène, Centre Hospitalier, 64000 Pau, France; 70 Service de réanimation néonatale, Centre Hospitalier, 62100 Calais, France; 71 Service de réanimation néonatale, Centre Hospitalier Universitaire, Kremlin Bicêtre, APHP, 94275 Le Kremlin Bicêtre, France; 72 Laboratoire de Microbiologie, Centre Hospitalier, 62300 Lens, France; 73 Équipe opérationnelle d’hygiène, Université de Reims Champagne-Ardenne, 51100 Reims, France; 74 Équipe opérationnelle d’hygiène, Centre Hospitalier, 95107 Argenteuil, France; 75 Équipe opérationnelle d’hygiène, Centre Hospitalier Universitaire, 14000 Caen, France; 76 Laboratoire de Biologie Médicale, Centre Hospitalier Métropole Savoie-Site de Chambéry, 73 011 Chambéry, France; 77 Laboratoire de Microbiologie, Centre Hospitalier, 64100 Bayonne, France; 78 Service de réanimation néonatale, Centre Hospitalier Universitaire, Hôpital de la Conception, APHM, 13005 Marseille, France; 79 Laboratoire de Microbiologie, Hôpitaux de Brabois, 54035 Nancy, France; 80 Laboratoire de Microbiologie, Centre Hospitalier, 79021 Niort, France; 81 Laboratoire de Microbiologie, Centre Hospitalier Universitaire, 97159 Pointe-à-Pitre, France; 82 Service de réanimation néonatale, Centre Hospitalier Universitaire Charles Nicolle, 76000 Rouen, France; 83 Laboratoire de Microbiologie, Centre Hospitalier, 95300 Pontoise, France; 84 Équipe opérationnelle d’hygiène, Centre Hospitalier Universitaire, 35000 Rennes, France; 85 Service de réanimation néonatale et réanimation pédiatrique, Centre Hospitalier Universitaire, Hôpital Robert Debré, Inserm UMR-S 1250 P3Cell, Université de Reims Champagne-Ardenne, 51100 Reims, France; 86 Laboratoire de Microbiologie, Centre Hospitalier Universitaire, 37044 Tours, France; 87 Équipe opérationnelle d’hygiène, Centre Hospitalier Universitaire Charles Nicolle, 76000 Rouen, France; 88 Équipe opérationnelle d’hygiène, Centre Hospitalier, 68100 Mulhouse, France; 89 Équipe opérationnelle d’hygiène, Centre Hospitalier Universitaire, 37044 Tours, France; 90 Service de réanimation néonatale, Centre Hospitalier du Havre, 76290 Montivilliers, France; 91 Laboratoire de Bactériologie, Université de Reims Champagne-Ardenne, 51100 Reims, France; 92 Service de réanimation néonatale, Centre Hospitalier, 68100 Mulhouse, France; 93 Laboratoire de Microbiologie, Centre Hospitalier, 62000 Arras, France; 94 Laboratoire de Microbiologie, Centre Hospitalier Universitaire de Martinique, 97261 Fort de France, France; 95 Service de réanimation néonatale, Centre Hospitalier Universitaire, 42055 Saint Etienne, France; 96 Laboratoire de Microbiologie, Centre Hospitalier, 72000 Le Mans, France; 97 Polyclinique Majorelle, 54000 Nancy, France; 98 Laboratoire de Microbiologie, Centre Hospitalier Universitaire, 42055 Saint Etienne, France; 99 Laboratoire de Microbiologie, Centre Hospitalier, 81100 Castres, France; 100 Service de réanimation néonatale, Centre Hospitalier, 72000 Le Mans, France; 101 Laboratoire de Microbiologie, Centre Hospitalier Universitaire, 06200 Nice, France; 102 Équipe opérationnelle d’hygiène, Centre Hospitalier Inter-Communal, 94010 Créteil, France; 103 Service de réanimation néonatale, Centre Hospitalier, 22000 Saint Brieuc, France; 104 Équipe opérationnelle d’hygiène, Centre Hospitalier Universitaire de Martinique, 97261 Fort de France, France; 105 Service de réanimation néonatale, Centre Hospitalier Universitaire, 14000 Caen, France; 106 Équipe opérationnelle d’hygiène, Centre Hospitalier, 62000 Arras, France; 107 Laboratoire de Microbiologie, Centre Hospitalier, 64000 Pau, France; 108 Service de réanimation néonatale, Centre Hospitalier Régional, 45100 Orléans, France; 109 Agence Santé Publique France, 94415 Saint Maurice, France

**Keywords:** *Staphylococcus capitis*, NRCS-A clone, Bloodstream catheter-related infection, Neonatal Intensive Care Unit (NICU), Preterm babies, Neonates, Nationwide active surveillance

## Abstract

To increase the knowledge about *S. capitis* in the neonatal setting, we conducted a nationwide 3-month survey in 38 neonatal intensive care units (NICUs) covering 56.6% of French NICU beds. We demonstrated 14.2% of *S. capitis* BSI (*S.cap*BSI) among nosocomial BSIs. *S.cap*BSI incidence rate was 0.59 per 1000 patient-days. A total of 55.0% of the *S.cap*BSIs were late onset catheter-related BSIs. The *S. capitis* strains infected preterm babies (median gestational age 26 weeks, median birth weight 855 g). They were resistant to methicillin and aminoglycosides and belonged to the NRCS-A clone. Evolution was favorable in all but one case, following vancomycin treatment.

## Introduction

Catheter-related bloodstream infections (CRBSI) are associated with increased rates of morbidity in intensive care unit patients and in neonates [1]. The prevention of the avoidable part of CRBSIs is a public health priority [2, 3]. In this context, since 2019, all French hospitals and clinics are encouraged to participate in an annual 3-month survey of CRBSI coordinated by the national infection control SPIADI network. Over the last two decades, multidrug-resistant *Staphylococcus capitis* has been increasingly reported as a major agent responsible for CRBSI in preterm babies [4]. Therapeutic failures likely due to heteroresistance to vancomycin in this bacteria [5] and local epidemics have been identified and investigated in NICUs [5–7]. *S. capitis* seems to be particularly well-adapted to the NICU environment, possibly in connection with its ability to produce biofilm [8, 9]. However, the neonate contamination routes remain obscure. Recent studies performed in distinct parts of the world have demonstrated a single lineage within the *S. capitis* species, named NRCS-A, responsible for invasive neonatal infections worldwide [10, 11]. The mechanisms that have driven the global dissemination of this clone have not yet been elucidated. We report the results of the 3-month nationwide BSI survey conducted during the first quarter of 2019 in the largest series of NICUs located in 38 French hospitals. We present clinical data related to the neonates suffering from BSI, and the incidence rates and major characteristics of the neonatal BSIs. In addition, using molecular methods, we characterized the isolates responsible for *S. capitis* BSIs to establish whether or not they belong to the NRCS-A clone. We provide new data that increase the knowledge about *S. capitis* in the current neonatal setting.

## Materials and methods

### BSI epidemiological survey method

#### Study population

**Fig. 1:**
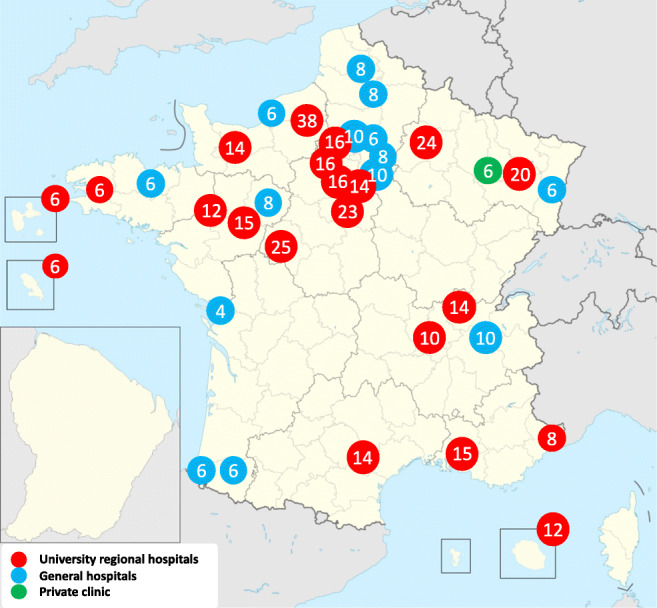
Location of the 38 participating centers and number of neonatal intensive care beds

Thirty-eight maternity hospitals comprising neonatal intensive beds participated in the study (Fig. 1). The 447 beds surveyed represented 56.6% of French neonatal intensive beds (https://www.data.gouv.fr/en/datasets/).

#### Study design

The surveillance program involved a 3-month survey of all cases of nosocomial BSI between January 1 and April 30 2019. The survey covered 33,971 intensive care patient-days (PD). Nosocomial BSIs were defined according to international definitions (CDC). The variables studied included clinical data (i.e., sex, gestational age, birth weight, death within 7 days of BSI diagnosis), major characteristics of the BSI such as the portal of entry (skin [primitive cutaneous form or superinfection of a skin breach], lungs, urine, intravascular device, or digestive tract), and for catheter-related BSI, the time lag between the insertion of the catheter, and the appearance of the clinical signs of the BSI. The BSI incidence rates were calculated per 1000 PD. Ethical approval of the surveillance program was obtained at the national level from the Réseau de Prévention des Infections Associées aux Soins.

#### Microbiological study

PFGE was used as a typing technique [12].

#### Statistical data

The data were analyzed with R software. Chi-square tests and Fisher’s exact test (two-tailed) were used to test associations, and a *P* value of 0.05 was considered significant.

## Results

### Epidemiology of neonatal BSI

During the study period, 141 nosocomial BSIs were diagnosed in 81 male and 60 female neonates. The mean BSI incidence rate was 4.15 per 1000 PD (Table 1). The most frequently isolated micro-organisms were *S. epidermidis* (39.0%), *S. aureus* (17.0%), *S. haemolyticus* (15.6%), and *S. capitis* (14.2%). Twenty BSIs were polymicrobial (14.2%).

**Table 1:** BSI, B-cvc, and B-uvc incidence rates per 1000 PD according to the participating centers

enters			BSI incidence rates per 1000 PD								
	During the 3-month survey		BSI					B-cvc			B-uvc
	PD	Nosocomial BSI	All	*S. aureus*	*S. epidermidis*	*S. capitis*	*Enterobacteriaceae*	All	*S. aureus* B-cvc	*S. capitis* B-cvc	All
Participating centers with a neonatal intensive care unit											
University regional hospitals											
1	2,443	10	4.09	0.82	2.45	0.41	0.00	1.64	0.41	0.41	2.46
2	1,840	7	3.80	1.09	0.54	0.00	1.63	1.63	0.54	0.00	0.54
3	1,825	10	5.48	2.19	1.64	0.00	0.55	0.00	0.00	0.00	0.00
4	1,658	14	8.44	2.41	4.22	0.60	0.60	3.01	1.21	0.60	0.60
5	1,482	6	4.05	0.67	0.00	2.02	0.00	1.35	0.67	0.67	0.00
6	1,332	8	6.01	0.00	3.00	1.50	1.50	3.00	0.00	0.75	1.50
7	1,322	10	7.56	0.76	3.02	0.76	0.76	0.00	0.00	0.00	0.00
8	1,204	0	0.00	0.00	0.00	0.00	0.00	0.00	0.00	0.00	0.00
9	1,134	8	7.05	0.00	2.64	1.76	0.00	3.53	0.00	0.88	0.88
10	1,114	0	0.00	0.00	0.00	0.00	0.00	0.00	0.00	0.00	0.00
11	1,062	0	0.00	0.00	0.00	0.00	0.00	0.00	0.00	0.00	0.00
12	1,023	6	5.86	0.00	2.93	0.98	0.00	0.98	0.00	0.98	1.95
13	1,016	3	2.97	0.98	0.98	0.98	0.00	0.98	0.00	0.00	0.98
14	999	3	3.00	0.00	2.00	0.00	0.00	1.00	0.00	0.00	0.00
15	892	4	4.48	0.00	1.12	2.24	1.12	2.24	0.00	1.12	1.12
16	822	0	0.00	0.00	0.00	0.00	0.00	0.00	0.00	0.00	0.00
17	764	5	6.54	1.31	3.93	1.31	0.00	2.62	0.00	0.00	0.00
18	793	5	6.31	1.26	2.52	1.26	0.00	3.78	0.00	1.26	0.00
19	636	0	0.00	0.00	0.00	0.00	0.00	0.00	0.00	0.00	0.00
20	545	4	11.00	0.00	0.00	0.00	1.83	5.50	0.00	0.00	1.83
21	524	0	0.00	0.00	0.00	0.00	0.00	0.00	0.00	0.00	0.00
General hospitals											
22	972	3	3.09	1.03	1.03	1.03	0.00	2.06	1.03	1.03	1.03
23	893	1	1.12	0.00	0.00	1.12	0.00	1.12	0.00	1.12	0.00
24	890	5	5.62	2.25	2.25	1.12	1.12	1.12	1.12	0.00	1.12
25	769	0	0.00	0.00	0.00	0.00	0.00	0.00	0.00	0.00	0.00
26	753	6	7.97	3.98	0.00	1.33	2.66	1.33	1.33	0.00	0.00
27	595	2	3.36	0.00	1.68	0.00	0.00	1.68	0.00	0.00	0.00
28	570	0	0.00	0.00	0.00	0.00	0.00	0.00	0.00	0.00	0.00
29	493	6	12.20	0.00	6.08	0.00	0.00	2.03	0.00	0.00	10.14
30	401	0	0.00	0.00	0.00	0.00	0.00	0,00	0.00	0.00	0.00
31	396	1	2.52	0.00	2.52	0.00	0.00	2.52	0.00	0.00	0.00
32	369	0	0.00	0.00	0.00	0.00	0.00	0.00	0.00	0.00	0.00
33	353	2	2.68	0.00	0.00	0.00	0.00	0.00	0.00	0.00	0.00
34	320	3	9.38	0.00	6.25	0.00	3.12	0.00	0.00	0.00	0.00
35	308	7	22.72	0.00	9.74	0.00	6.49	9.74	0.00	0.00	0.00
36	275	2	7.27	3.64	0.00	0.00	3.64	3.64	3.64	0.00	0.00
Participating centers with intensive care beds in neonatal medical unit											
General hospital											
22	854	0	0.00	0.00	0.00	0.00	0.00	0.00	0.00	0.00	0.00
Private clinic											
40	330	0	0.00	0.00	0.00	0.00	0.00	0.00	0.00	0.00	0.00
All	33,971	141	4.15	0.71	1.62	0.59	0.50	1.38	0.26	0.29	0.68

The portal of entry of the BSIs was suspected or proven in 83.7% of the cases. The digestive tract (12.1%), the skin (8.5%), and the pulmonary tract (6.4%) were minor portals of entry. Most of the BSIs were catheter-related (70 CRBSIs; 50.0 %) (Table 2). The CRBSI involved a central venous catheter (CVC) in 47 cases (67.1%), all but one associated with *staphylococci* (97.9%), and an umbilical venous catheter (UVC) in 23 cases (32.9%). The UVC-related BSIs were more diverse than those related to CVC: *enterococci-*, *Enterobacteriaceae-*, and *B. cereus-*BSIs were more frequent with UVC-BSIs (26.1%) rather than with CVC-BSIs (4.3%) (*p* = 0.022). The median time lag between the insertion of the catheter and the appearance of the clinical signs of the BSI was significantly longer for *S. capitis* (63.6%, ≥ 10 days) rather than for *S. aureus* (7.7%), *S. epidermidis* (16.1%), *S. haemolyticus* (30.8%), *enterococci*, and *Enterobacteriaceae* (no case) (*p* = 0.018; Table 3).

**Table 2:** Major characteristics of the BSIs and infected neonates according to the micro-organism

	BSIs											Infected neonates				
	*N*	Portal of entry								Sex		Birth weight (g)		Gestational age (week)		Early death (%)
		CVC	UVC	Cutaneous	Pulmonary	Urinary	Digestive	Others	Not identified	Male	Female	< 1500 g	Median	< 33 weeks	Median	
Micro-organism																
All	141	47	23	12	9	1	17	9	23	81	60	112 (79.4)	980	113 (80.1)	28	22 (15.6)
*S. aureus*	24	9	4	4	4		1	1	1	12	12	16 (66.7)	1,100	16 (66.7)	30	7 (29.2)
*S. epidermidis*	55	20	11	6	1		4	1	12	35	20	43 (78.2)	910	43 (78.2)	27	5 (9.1)
*S. haemolyticus*	22	10	3		2		3	1	3	10	12	22 (100.0)	917	21 (95.4)	27	3 (13.6)
*S. capitis*	20	10	1	1	1		3		4	12	8	16 (80.0)	855	15 (75.0)	26	1 (5.0)
*Enterococci*	7	1	3				1	1	1	6	1	4 (57.1)	1,260	4 (57.1)	31	1 (14.3)
*Enterobacteriaceae*	17	1	2		2	1	4	5	2	9	8	9 (52.9)	1,480	11 (64.7)	29	5 (29.4)
*Bacillus cereus*	3		1	1					1	0	3	2 (66.7)	745	3 (100.0)	28	0

**Table 3:** Time lag between the insertion of the catheter and the appearance of the clinical signs of the CRBSI

	Number of CRBSIs	Time lag (days)			
		Mean	Median	< 10 days	≥ 10 days
Micro-organism					
*S. aureus*	13	7.2	6	11	3
*S. epidermidis*	31	8.0	6	26	5
*S. haemolyticus*	13	8.1	6	9	4
*S. capitis*	10	10.3	10	4	7
*Enterococci*	4	6.2	6	4	0
*Enterobacteriaceae*	3	4	4	3	0

### Characteristics of the infected neonates

The gestational age of the infected neonates ranged between 24 and 41 weeks (median value 28), and their birth weight ranged between 455 and 4050 g (median value 1100); 15.6% of the neonates died during the 7-day period after the diagnosis of the BSI. BSIs involving *S. aureus*, *Enterobacteriaceae*, and *Enterococci* were associated with the highest prevalence of early death among infected neonates (29.4, 29.2, and 14.3% for *Enterobacteriaceae-*, *S. aureus-*, and *Enterococci*-associated BSIs, respectively). The prevalence of BSI in the neonates with the a gestational age ≥ 33 weeks and a birth weight > 1500 g differed according to the bacteria (Table 2): it was the highest for *Enterococci* (42.9%), *Enterobacteriaceae* (35.3%), and *S. aureus* (29.2%), lower for *S. capitis* (20.0%) and *E. epidemidis* (18.2%) and nil for *S. haemolyticus* and *B. cereus* (*p* = 0.056).

### *S. capitis* BSI characteristics and antibiotic susceptibility of *S. capitis* strains

Twenty BSIs were associated with *S. capitis* (14.2%), resulting in a mean incidence of 0.59 per 1000 PD, ranging between 0 and 2.24 according to centers (Table 1); 39.5% of the NICUs reported at least one *S. capitis*-BSIs. The *S. capitis*-BSIs were significantly associated with the largest NICUs: at least one *S. capitis*-BSIs was reported in 15 of the 22 NICUs with ≥ 10 beds, whereas none was reported in the 14 NICUs with < 10 beds (*p* < 0.001). Four NICUs documented two (*n* = 3) or three (*n* = 1) *S. capitis*-BSIs during the survey period. The antibiotic susceptibility patterns of 18 strains were available (90.0%). Most of the strains were resistant to multiple antibiotics, i.e., methicillin (100%), gentamicin (100%), rifampicin (61.1%), fosfomycin (55.5%), erythromycin (44.4%), fluoroquinolones (33.3%), and fusidic acid (22.2%). Vancomycin and teicoplanin MIC values ranged between 0.25 and 4 mg/L (Table 4). Data regarding antibiotic treatment were available for 18 cases: 17 neonates received vancomycin over 2–24 days (median value: 8 days) and the remaining neonate received linezolid (11 days). A favorable outcome was observed in all but one case. An early death was observed for a preterm infected neonate (gestational age 25 weeks; birth weight 455 g), who received vancomycin over 3 days following the detection of a *S. capitis* and *S. haemolyticus*-associated CRBSI.

**Table 4:** Antibiotic susceptibility of the *S*. *capitis* strains

Centers	Strain	Antibiotype*	MIC vancomycine (mg/L)	MIC teicoplanine (mg/L)
1	1	Oxa KTG Ri Fu	0.5	< 0.25
9	2	Oxa KTG Ri Fo	0.5	< 0.25
	3	Oxa KTG Ri Fo	0.5	< 0.25
4	4	Oxa KTG Ri Fo	–	–
7	5	Oxa AKTG Ri Fu Ery	–	–
13	6	Oxa TG Nor	1	2
6	7	Oxa G Cip Ery Ri	< 4	< 2
	8	Oxa G Cip Ery	< 4	< 2
5	9	Oxa ATG Ri Fo Te(I) Ery(I) Pr(I)	1	0.5
	10	Oxa ATG Ri Fo Te(I) Ery(I) Pr(I)	1	0.5
	11	Oxa ATG Ri Fo Te(I) Ery(I) Pr(I)	1	0.5
15	12	Oxa ATG Cip Fo	1	2
	13	Oxa ATG Cip Fo	1	1
17	14	Oxa AKTG Cip Fo	1	1
12	15	Oxa KTG Ery	2	4
22	16	Oxa AKTG	0.5	< 0.25
18	17	Oxa ATG Ri Fu	0.5	< 0.25
26	20	Oxa ATG Ri Fo Te(I) Ery(I) Pr(I)	1	2

*Oxa* oxacillin, *K* kanamycin, *T* tobramycin, *G* gentamicin, *A* amikacin, *Ri* Rifampicin, *Fu* fusidic acid, *Fo* fosfomycin, *Te* tetracyclin, *Ery* erythromycin, *Pr* pristinamycin, *Nor* norfloxacin, *Cip* ciprofloxacin

**Fig. 2:**
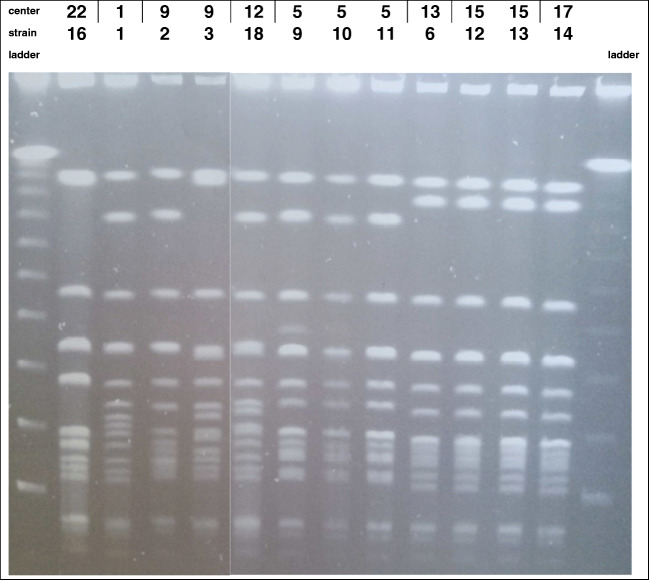
*Sma*I PFGE patterns of the *S. capitis* strains responsible for neonatal BSI

Twelve *S*. *capitis* BSI strains from 8 NICUs were available for molecular typing. A considerable homogeneity was demonstrated among the strains, and PFGE pattern analysis demonstrated that all strains belonged to the NRCS-A clone [10] (Fig. 2). Regarding the three NICUs that reported several *S*. *capitis*-BSI cases, the strains isolated in a same center shared the same pattern in two cases. In addition, the strains isolated from three distinct centers located in two distant French regions shared the same pattern.

## Discussion

This nationwide study adds several elements to the available data on *S. capitis* responsible for neonatal BSI*.*

We provide a first mean incidence of *S. capitis* BSIs in French NICUs*. S. capitis* BSIs currently involve an average of one neonate per 1700 PD, which is lower than that observed for *S. aureus* and *S. epidermidis*, but higher than that of *Enterobacteriaceae* in the population of neonates surveyed*.* Our findings confirm *S. capitis* as a significant agent responsible for nosocomial BSI in the neonatal setting [10, 11, 13]*.*

Second, such as *S. epidermidis* and *S. haemolyticus*, we showed that *S. capitis* preferentially infects the more fragile neonates and thus confirmed that *S. capitis* is an opportunistic pathogen, devoid of great virulence potential*.* Concordant with previous studies [13], all the *S. capitis* strains responsible for BSIs displayed resistance to methicillin and gentamicin, but remained susceptible to vancomycin*. S. capitis*-BSIs have been taken into account by the clinicians, and vancomycin probably played a crucial role in the recovery of neonates*.*

Third, we identified one particularity distinguishing *S. capitis* among the bacteria associated with CRBSI cases. Our study reveals a doubled lag time between insertion of the catheter and the first signs of the BSI involving *S. capitis* when compared with other bacteria*.* The absence of early infection likely excludes a contamination of the catheter at the time of its insertion, but rather indicates that the contamination of the catheter may have occurred following catheter manipulations among neonates presenting the longest periods of catheterization*.*

Finally, the molecular analysis of a large part of the *S. capitis* strains indicates that they belong to the multidrug-resistant NRCS-A clone and highly suggests likely epidemic phenomena among the NICUs presenting the highest incidence rates of *S. capitis* BSIs*.*

## Conclusion

Our data confirm the clone NRCS-A particularly well-suited to the neonatal setting and its cumbersome epidemiology [10, 11, 13]*.* In most NICUs, *S. capitis* BSIs remain relatively infrequent among neonates, but concern primarily the most fragile ones. In order to better determine the factors involved in the occurrence of these infections, monitoring of BSIs should be continued and complemented by a systematic investigation when several cases are identified over a 3-month period in the same NICU*.*
